# Osthole improves collagen-induced arthritis in a rat model through inhibiting inflammation and cellular stress

**DOI:** 10.1186/s11658-018-0086-0

**Published:** 2018-05-02

**Authors:** Renguo Xu, Zhen Liu, Jiande Hou, Tao Huang, Ming Yang

**Affiliations:** 1 Department of Osteology, YeDa Hospital, Taishan Road No. 11 Economic and Technological District of Yantai, Yantai, 264006 China; 2 School of Pharmacy, Jiangsu university, Zhenjiang, 212000 China

**Keywords:** Osthole, Collagen-induced arthritis, Synovial fibroblasts, Inflammation, NF-κB

## Abstract

**Background:**

Osthole is a natural product that has multiple bioactive functions and has been reported to exert potent immunosuppressive effects. However, the therapeutic effect of osthole on arthritis has not been explored. In the present study, a collagen-induced arthritis rat model, IL-1β-stimulated SW982 cells, and RA-like fibroblast-like synoviocytes (FLS) were employed to investigate the effect and possible mechanism of osthole on arthritis in vivo and in vitro.

**Results:**

20 and 40 mg/kg osthole significantly alleviated collagen-induced arthritic symptoms based on histopathology and clinical arthritis scores, and improved erosion using HE staining. 20 and 40 mg/kg osthole decreased the level of IL-1β, TNF-α and IL-6 in rats and ameliorated oxidative stress in serum evaluated using ELISA kits. In addition, treatment with 50 and 100 μM osthole for 48 h inhibited 10 ng/ml IL-1β-stimulated proliferation and migration of SW982, and significantly inhibited the expression of matrix metalloproteinases, such as MMP-1, MMP-3 and MMP-13, as detected by western blot. 50 and 100 μM osthole also blocked the generation of IL-6 and TNF-α in IL-1β-stimulated SW982 cells. The NF-κB and MAPK pathways were also inhibited by osthole in IL-1β-treated SW982 cells.

**Conclusion:**

These results collectively demonstrated that osthole improves collagen-induced arthritis in a rat model and IL-1β-treated SW982 cells through inhibiting inflammation and cellular stress in vivo and in vitro, and osthole might be a promising therapeutic agent for RA.

## Introduction

Rheumatoid arthritis (RA) is a common chronic inflammatory autoimmune disease that is characterized by chronic inflammation, hyperplasia of synovial tissue and erosion of cartilage and bone [1]. Almost all RA patients suffer the loss of joint function, which results in serious social problems and an economic burden. The pathogenesis of RA is a complicated process involving genetic variants, environmental factors and immune system responses [2–4].

Fibroblast-like synoviocytes (FLS), a vital component of synovial tissue, play an important role in the process of joint destruction through the secretion of various cytokines, proteases and arachidonic acid metabolites [5]. Activation of FLS contributes to RA through damaging synovial membranes and producing inflammatory factors to recruit immune cells. Some studies have shown that synovial fibroblasts are one of the key effector cells of RA and the excessive proliferation and invasion of synovial fibroblasts play a critical role in the pathogenesis of RA [6, 7]. Blocking FLS activation to reduce activating cytokines using small molecules has been a promising RA therapeutic method [8]. Therefore, synovial fibroblasts have become a unique target cell for studying RA pathogenesis and treatment.

**Fig. 1:**
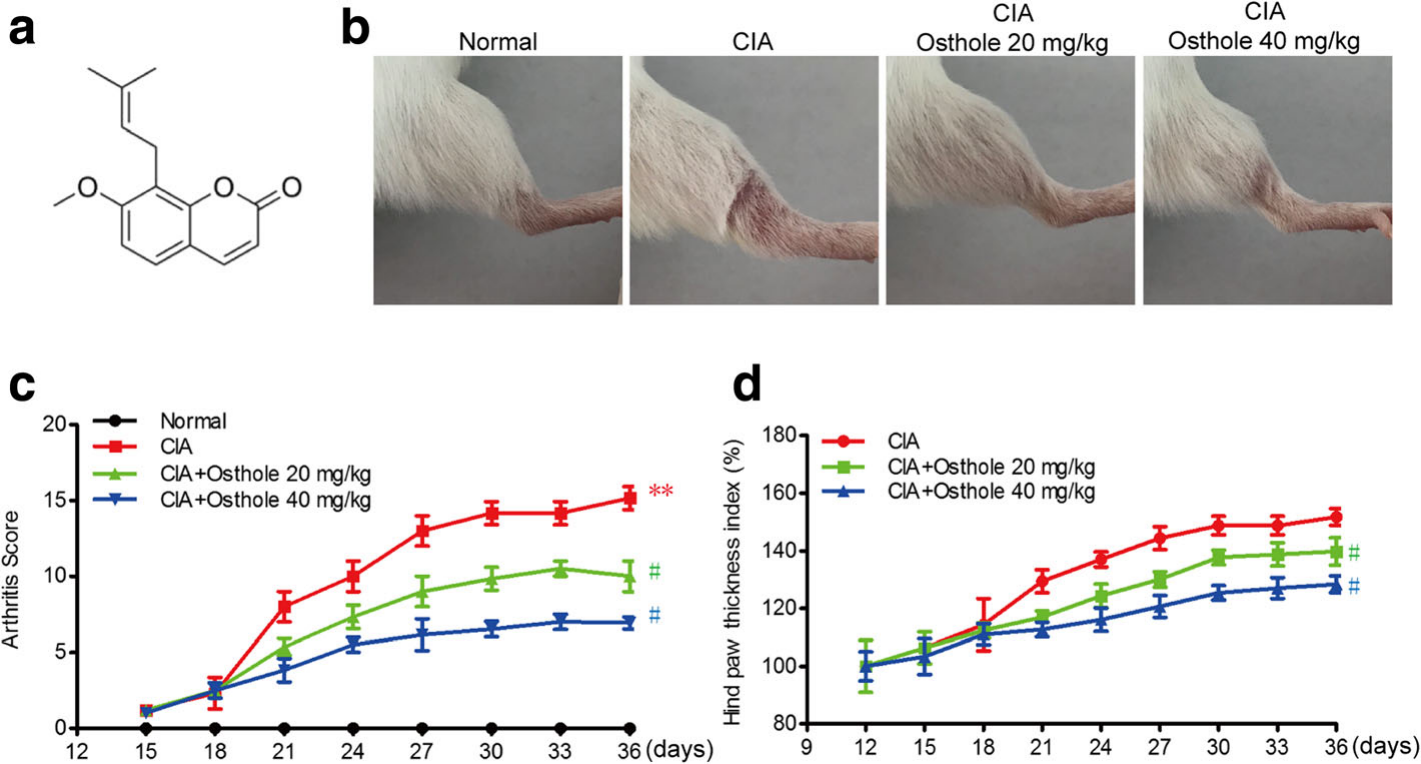
Osthole attenuates collagen-induced arthritis in a rat model. **a** Structure of osthole. **b** Representative pictures of ankle joint swelling in different groups. **c** Rats were orally administered osthole (20 and 40 mg/kg) or normal saline from day 12 after the first immunization. The arthritis severity score was calculated from day 15 until sacrifice. **d** The hind paw thickness was evaluated from day 12 until sacrifice. **p < 0.01 compared with normal group, ^#^*p* < 0.05 compared with CIA group

Osthole (Fig. 1a), the main coumarin extracted from *Cnidium monnieri* (L.) Cusson, exhibits wide pharmacological effects, such as anti-cancer and anti-inflammatory effects and cardiovascular protection [9–11]. Osthole blocks NF-κB activation to protect against ovalbumin-induced asthma in a murine model [12]. In addition, osthole markedly reduced NF-κB and mitogen-activated protein kinase (MAPK) cascades to ameliorate acute myocardial infarction in rats [13]. Osthole decreases reactive oxygen species generation and NF-κB-mediated COX-2 expression [14]. Moreover, osthole can also inhibit the invasive ability through suppression of NF-κB-mediated MMP-9 expression [15]. However, no studies have explored the protective effect of osthole in a collagen-induced arthritis rat model. In the present study, we assessed the effects of osthole on CIA in a rat model with respect to its possible mechanism by determining the inflammatory factors and oxidative stress in vivo. We further revealed the anti-proliferation, anti-inflammatory and anti-cellular stress mechanisms of osthole using IL-1β-treated SW982 cells in vitro.

## Materials and methods

### Reagent

Osthole was obtained from Aladdin (Aladdin, Shanghai, China). Sodium dodecyl sulfate (SDS), ammonium persulfate (APS), TritonX-100, Tris base, glycine, hematoxylin and eosin were purchased from Sigma Chemical Co (St Louis, MO, USA). Bovine type II collagen was bought from Chondrex (Chondrex, Redmond, WA). Tetramethylethylenediamine (TEMED) and Tween 20 were purchased from Thermo Fisher Scientific (Waltham, MA). 30% Acrylamide/Bis Solution (37.5:1) and loading buffer were obtained from Bio-Rad Laboratories (Richmond, Calif., USA). Pre-stained protein marker was obtained from Fermentas (Glen Burnie, MD, USA). PVDF membrane was purchased from Millipore (Bedford, MA, USA). Antibodies to IKKα (11930), IKKβ (#8943), p-IKKα/β (#2697), IκBα(#4814), p-IκBα(#2859), p65 (#8242), p-p65 (#3033), SAPK/JNK (#9295), p-SAPK/JNK (#4668), Erk1/2 (#4695), p-Erk1/2 (#4370), p38 (#8690), p-p38 (#4511), β-actin (#5174), goat anti-rabbit horseradish peroxidase (HRP) (#7074) and RIPA cell lysis buffer (#9806) were purchased from CST (Boston, MA).

### Experimental group and collagen-induced arthritis

Collagen-induced arthritis (CIA) is a common autoimmune animal model used to study rheumatoid arthritis (RA). Osthole was dissolved in 0.5% sodium carboxymethylcellulose (CMC-Na) solutions (final concentration 4 mg/ml). 150–180 g male Sprague-Dawley rats were divided into four experimental groups (*n* = 8/group): the normal group (0.5% CMC-Na), collagen-induced arthritis (CIA) group (0.5% CMC-Na), 20 mg/kg osthole + CIA group and 40 mg/kg osthole + CIA group. All animal experiments were performed according to the Helsinki Declaration and with approval of the Animal Experimentation Ethics Committee of YeDa Hospital. Type II collagen (2 mg/ml) was sufficiently emulsified with an equal volume of complete Freund’s adjuvant on ice. The rats were subjected to intradermal multi-point administration of 0.2 ml emulsified collagen (200 mg) at the base of the tail. A booster injection was given on day 10 to induce a high incidence and severity of arthritis. Collagen-incomplete Freund’s adjuvant emulsion was prepared and 0.1 ml (100 mg) of the emulsion was injected subcutaneously into the tail. The initiation of arthritis occurs within 1 week after the enhanced immunization. The rats were monitored for the progression and severity of paw inflammation after the booster administration. The first immunization day was defined as day 0. Hind paw thickness was determined with electronic digital calipers (Shandong Tools Manufacturing Co., Shandong, China) every 3 days, starting from day 12. The calculated paw thickness of the CIA group and CIA + osthole group was compared to that of the Normal group. On day 15 after the first immunization, rats displayed the onset of arthritis and were randomly divided into groups. Osthole treatment was started on day 12 and continued for 24 days until day 36.

### Clinical assessment of arthritis

Macroscopic signs of clinical arthritis were assessed by a qualitative clinical score every 3 days beginning on day 15 when arthritic signs were first visible. Each paw was scored according to the slightly modified criteria [16]: 0, normal; 1, mild redness and swelling of ankle or wrist joints; 2, moderate redness and swelling of ankle or wrist joints; 3, severe redness and swelling of the entire paw including digits; and 4, paws with deformity or ankylosis. The maximum score for a single paw was 4 and for a single rat was 16.

### Histopathological assessment

Hind limbs were dissected on day 42, and the tissues were immediately placed in 4% paraformaldehyde for 24 h and then decalcified in 10% EDTA for 2 months at 4 °C and embedded in paraffin. Four-micrometer tissue sections were stained with hematoxylin and eosin (HE). Articular synovial membrane degeneration and proliferation, inflammatory cell infiltration and granulation tissue formation, the reduction or occlusion of the articular cavity and the damaged or fibrotic articular cartilage were evaluated according to the severity of the lesion in each part of the joint. The rating criteria for studies on synovial hyperplasia are as follows: 0 = no hyperplasia, 1 = inflammatory cell infiltration, 2 = mild hyperplasia, 3 = moderate hyperplasia and 4 = severe hyperplasia.

### Cell viability

Osthole was dissolved in DMSO (final concentration 200 mM). Cells of the SW982 synovial cell line were seeded into a 96-well culture plate with the density of 5 × 10^3^ per well and were treated with 0.1% DMSO as the control and 12.5, 25, 50, 100, 200 μM osthole for 24 h and 48 h. SW982 synovial cells were seeded into a 96-well culture plate with the density of 5 × 10^3^ per well and were treated together with 10 ng/ml IL-1β and osthole for 48 h. 100 μl/well of MTT (1 mg/ml, dissolved in PBS solution) was added into the 96-well plates. After 4 h incubation at 37 °C, 100 μl of DMSO was also added. One hour later, the absorbance was determined using a multiplate reader at a wavelength of 570 nm.

### Cell migration

For the migration assay, SW982 cells were treated with 10 ng/ml IL-1β and different concentrations of osthole for 24 h. The cells were plated into the top chamber of Transwells containing 8-μM diameter pores in 200 μl serum-free DMEM in triplicate. Medium containing 10% FBS was added to the lower chamber. After 12 h of incubation, cells remaining on the upper chamber were removed by cotton swab scrubbing. Cells on the lower surface of the membrane were fixed in 10% formalin at room temperature for 30 min and stained with 0.5% crystal violet.

### Western blot analysis

SW982 cells undergoing different treatments were collected and lysis buffer containing protease cocktail inhibitors (Roche) was added. The homogenate was centrifuged at 14000 g for 15 min at 4 °C and the protein concentration determined using a BCA kit. Whole cell lysate (20 μg) was loaded onto 12.5% SDS-PAGE. Electrophoresis was performed using a stacking gel at 80 V for 20 min and a separating gel at 110 V for 70 min. The proteins were transferred to PVDF membranes (Millipore, MA, USA) using an electro-blotting apparatus (Bio-Rad, CA, USA) at 300 mA for 90 min. The membranes were blocked for 1 h in TBST containing 0.1% Tween-20 and 5% dry milk and then incubated overnight with primary antibodies. After washing 3 times in TBST, the membrane was incubated for 2 h with horseradish peroxidase-conjugated secondary antibodies. The optical densities of the antibody-specific bands were analyzed using a Luminescent Image Analyzer (Alpha, USA).

### Determination of oxidative stress

iNOS, GPx, MDA and SOD assay kits were obtained from the Institute of Biological Engineering of Nanjing Jiancheng (Nanjing, China) and were used to determine the oxidative stress in rat plasma.

### Elisa

An ELISA kit (Dakewei, China) was used for quantitative measurement of inflammatory factors, such as IL-1β, TNF-α and IL-6, according to the manufacturer’s recommendations.

### Statistical analysis

The experimental data were expressed as mean ± S.D. of at least three independent experiments in cell experiments. Statistical analysis was performed using one-way analysis of variance (ANOVA) with the SPSS 18.0 statistical software. *P* values less than 0.05 were considered statistically significant.

## Results

### Osthole attenuates collagen-induced arthritis in the rat model

In order to determine the possible therapeutic effect of osthole on arthritis in vivo, the collagen-induced arthritis rat model was employed. The onset of arthritis in rats occurs within 7 days after the booster injection. Osthole treatment began on day 12 after the first immunization and continued until day 36. Clinical features, such as swollen red paws, malformation and functional impairment, were observed (Fig. 1b). Arthritis started to develop and worsen over time. As shown in Fig. 1c, CIA rats developed severe arthritis and reached a high score between days 27–36, as assessed by the clinical arthritic score. CIA rats treated with osthole showed a statistically significant progressive decrease in the severity of arthritis. The hind paw thickness was calculated using electronic digital calipers between days 12–36 (Fig. 1d). 20 mg/kg and 40 mg/kg osthole can significantly decrease hind paw thickness in CIA rats. These data demonstrate that osthole relieved swollen joints in CIA rats.

### Osthole inhibits inflammation and oxidative stress in the CIA model

**Fig. 2:**
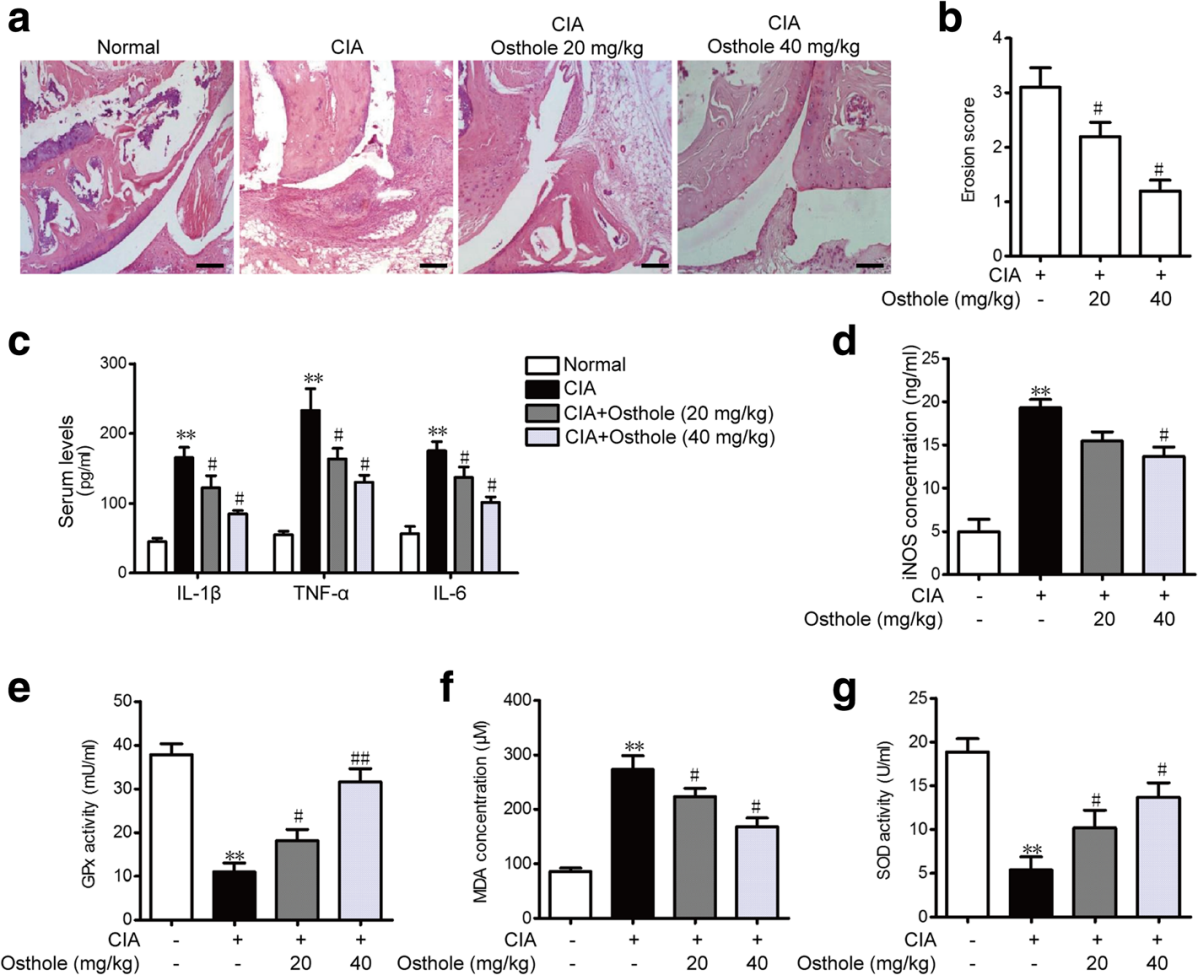
Osthole inhibits inflammation and oxidative stress in collagen-induced arthritis rat model. **a** Histological analysis was performed using rat hind paw sections stained with HE (scale bars 50 μm). **b** Histopathologic severity was assessed and calculated. **c** Levels of IL-1β, TNF-α and IL-6 in serum were measured by ELISA. Serum levels of several oxidative stress indicators, including iNOS concentrations (**d**), GPx activities (**e**), MDA concentrations (**f**) and SOD activities (**g**), were determined by assay kits. Values are expressed as the mean ± SD. **p < 0.01 compared with the normal control group, ^#^*p* < 0.05, ^##^*p* < 0.01 compared with the CIA group

Inflammatory cell infiltration, synovial hyperplasia and cartilage destruction were observed in histopathological observation (Fig. 2a). 40 mg/kg osthole improved the CIA, where infiltrated inflammatory cells, synovial hyperplasia and cartilage destruction were significantly decreased. The erosion scores were calculated as described above. 20 and 40 mg/kg osthole decreased the scores (Fig. 2b). A series of proinflammatory factors such as IL-1β, TNF-α and IL-6 were measured in rat plasma. As shown in Fig. 2c, the contents of IL-1β, TNF-α and IL-6 were elevated in CIA rats and osthole dramatically decreased these factors. The level of oxidative stress in serum, indicated by iNOS, GPx, MDA and SOD, was further detected. As depicted in Fig. 2d-g, CIA models had higher levels of oxidative stress while osthole decreased iNOS concentration and MDA concentration and increased GPx and SOD to protect against oxidation. These results indicated that osthole can improve synovial hyperplasia, inhibit inflammation and exert an anti-oxidation effect in CIA.

### Osthole alleviates IL-1β-induced proliferation and migration in the SW982 human synovial cell line

**Fig. 3:**
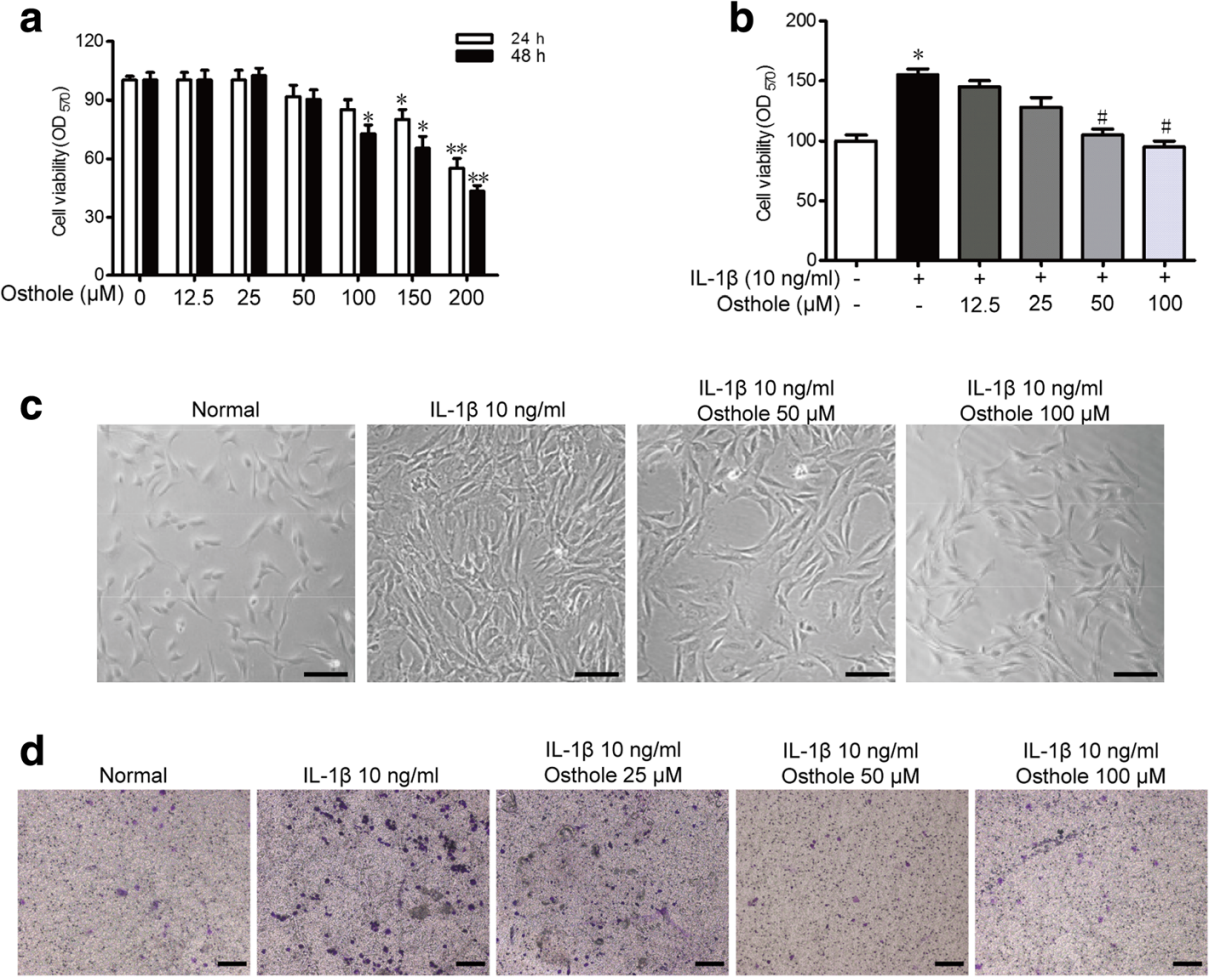
Osthole alleviates IL-1β-induced proliferation and migration in SW982 human synovial cell line. **a**, **b** To evaluate the effect of osthole on SW982 survival and on IL-1β-treated SW982 cells, SW982 cells were incubated with the indicated concentration of osthole (0–200 μM) alone for 24 and 48 h (A) or together with 10 ng/ml IL-1β in 96-well plates for 48 h. MTT assay was applied to measure SW982 survival. **c** Morphology of normal SW982 cells, 10 ng/ml IL-1β treated SW982 cells and 10 ng/ml IL-1β treated SW982 cells incubated with 50, 100 μM osthole for 48 h. **d** SW982 cells were incubated with IL-1β and osthole for 48 h and migration of SW982 cells was detected using Transwell assay. Representative images of crystal violet-stained migratory SW982 on the membrane. *p < 0.05, **p < 0.01 compared with the normal group, ^#^*p* < 0.05, ^##^*p* < 0.01 compared with the 10 ng/ml IL-1β group

At present, the ideal source of SF is the synovial tissue of advanced RA patients undergoing joint replacement surgery. However, most RA patients are mainly treated with drugs, so the human synovial sarcoma cell line (SW982) was applied to explain the protective mechanism of osthole in vitro. To investigate the therapeutic effects of osthole on SW982 cells, we first measured its potential cytotoxicity on SW982 cells in vitro. SW982 cells were incubated with different concentrations of osthole (12.5, 25, 50, 100, 150 and 200 μM) for 24 and 48 h, and cell viability was determined using the MTT assay. The results illustrated that 50 μM osthole for 48 h had no effect on the SW982 cell viability (Fig. 3a). Furthermore, the IL-1β-induced SW982 cells were used to mimic RA-SF in vitro. Cell proliferation was further detected by MTT. As shown in Fig. 3b, treatment with IL-1β for 48 h dramatically enhanced the proliferation and cell viability, which revealed that inflammatory factors in the microenvironment promote the proliferation of synovial fibroblasts. 50 and 100 μM osthole significantly suppressed IL-1β-induced proliferation. The cell morphology is exhibited in Fig. 3c. Migration of synovial fibroblasts is also involved in the progression of RA. Thus, the Transwell assay was applied to measure the migration of SW982. As exhibited in Fig. 3d, IL-1β significantly increased migration and 50 and 100 μM osthole significantly blocked migration. These results indicated that osthole can suppress IL-1β-induced proliferation and migration of SW982.

### Osthole suppresses IL-1β-induced expression of MMPs and pro-inflammatory factors in SW982 cells

**Fig. 4:**
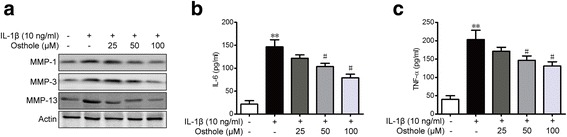
Osthole suppresses IL-1β-induced expression of MMPs and pro-inflammatory factors in SW982 cells. SW982 cells were cultured with 10 ng/ml IL-1β and different concentrations of osthole for 48 h. **a** Total proteins (MMP-1, MMP-3 and MMP-13) were extracted from the SW982 cells and analyzed by western blot. β-Actin was chosen as a loading control. **b** Culture supernatants were collected, and the cytokine concentrations of IL-6 (B) and TNF-α (**c**) in the medium were measured by ELISA kits. **p < 0.01 compared with the normal group, ^#^*p* < 0.05 compared with the 10 ng/ml IL-1β group

Activated synovial fibroblasts can secrete MMPs to rebuild cartilage. It is also observed that SW982 cells produced inflammatory cytokines and MMPs in response to IL-1β during RA. IL-1β significantly increased MMP-1, MMP-3 and MMP-13 expression, and osthole treatment for 48 h reduced the expression of MMP-1, MMP-3 and MMP-13 in a dose-dependent manner (Fig. 4a). The levels of pro-inflammatory factors, IL-6 and TNF-α, in the supernatants of SW982 cultures were measured using ELISA. As depicted in Fig. 4b and c, the stimulation of IL-1β significantly promoted the generation of IL-6 and TNF-α. Osthole treatment alleviated IL-1β-triggered release of IL-6 and TNF-α in SW982. Therefore, our results indicated that osthole may exert an anti-inflammatory effect on IL-1β-treated SW982 cells.

### Osthole inhibits IL-1β-induced activation of NF-κB and MAPK pathway

**Fig. 5:**
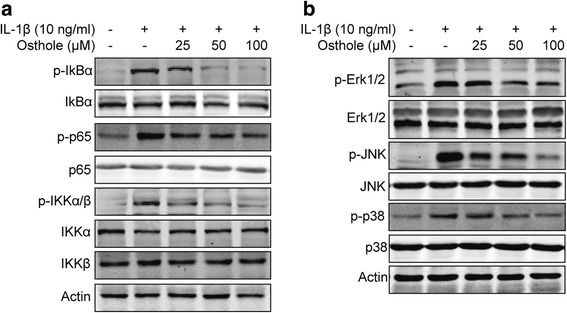
Osthole inhibits IL-1β-induced activation of NF-κB and MAPK pathway. **a** SW982 cells were treated with 25, 50 and 100 μM osthole and 10 ng/ml IL-1β for 48 h. The levels of IκBα, p65, IKKαβ and phosphorylated levels were detected by immunoblotting. β-Actin was chosen as a loading control. The result is representative of three experiments. **b** SW982 cells were treated with 25, 50 and 100 μM osthole and 10 ng/ml IL-1β for 48 h. The levels of ERK1/2, JNK and p38 and phosphorylated levels were detected by immunoblotting. β-Actin was chosen as a loading control. The result is representative of three experiments

NF-κB is a critical transcription factor for the induction of various proinflammatory factors. In order to gain an insight into osthole’s mechanism, we used Western blot to measure the proteins involved in the NF-κB signal pathway. 10 ng/ml IL-1β treatment for 48 h significantly increased phosphorylated IκBα, p65 and IKKα/β, which means IL-1β promoted activation of the NF-κB signal pathway. IκBα, p65 and IKKα/β phosphorylation were suppressed by the addition of osthole (25, 50, 100 μM) for 48 h (Fig. 5a). The MAPK signaling pathway is associated with inflammation and cellular stress. As shown in Fig. 5b, IL-1β treatment for 48 h can also significantly increase phosphorylated Erk1/2, JNK and p38 proteins. Osthole can decrease the MAPK signal in a dose-dependent manner. These results indicated that osthole exerted anti-inflammation and anti-proliferation effects via lowering phosphorylated proteins involved in NF-κB and MAPK signaling.

## Discussion

Collagen-induced arthritis is similar to rheumatoid arthritis in clinical symptoms and pathology, such as synovium hyperplasia, pannus formation and cartilage destruction. Therefore, CIA is the ideal and the most commonly used animal model to study RA. Synovial cell hyperplasia, a variety of inflammatory cell infiltration and pannus formation are the main pathological characteristics of RA, which in turn caused the destruction of the cartilage and bone tissue, eventually leading to joint deformity and the loss of function. In the CIA rat model, we observed that osthole can improve swollen joints and ankles.

The etiology and pathogenesis of RA are not yet clear, and RA is a typical multi-factor induced autoimmune disease. Activated T lymphocytes, B lymphocytes and macrophages play an important role in the pathogenesis of RA. IL-1β and TNF-α are the main inflammatory cytokines in the body, which play an important role in the pathogenesis of RA or experimental arthritis synovitis. IL-1β generation is mainly mediated by the NLRP3 inflammasome, and deletion of Nlrp3, caspase-1 and the IL-1 receptor markedly protects against rheumatoid-arthritis-associated inflammation [17]. IL-1β is the initiating factor in inflammation to regulate a variety of cytokines, cell adhesion molecules and inflammatory mediators, which are associated with bone erosion and cartilage destruction in RA. On one hand, excessive secreted IL-1β is able to stimulate the synthesis and release of PGE2 and collagenase from synovial cells and chondrocytes, resulting in a synovial inflammatory response and disintegration of cartilage matrix [18]. On the other hand, IL-1β can also act on endothelial cells to promote the aggregation of neutrophils, macrophages and lymphocytes, leading to aggravation of the inflammatory response in local joints. Apart from the release of inflammatory factors, proliferation of fibroblasts and tissue damage, TNF-α is also an activation factor of osteoclasts, which promotes the generation of osteoclasts and increases the activity of osteoclasts to cause cartilage destruction directly. In addition, TNF-α and IL-1β can trigger NF-κB activation in rheumatoid arthritis and infectious diseases [19]. Moreover, inflammation and oxidative stress are involved in the development and progression of rheumatoid arthritis (RA). Thus, we measured the levels of inflammatory factors and indicators of oxidative stress from CIA models, and found that osthole can reduce inflammation and stress.

Besides the immune system, fibroblast-like synoviocytes (FLS) are involved in various stages of RA disease [20]. Abnormal activated RA-FLS show active proliferation and have anti-apoptosis properties, which are the main cause of synovial tissue hyperplasia and also observed in HE staining. Various antigens trigger the immune response by identifying toll-like receptors on the surface of RA-FLS to activate the downstream signaling pathway. The activated RA-FLS release chemokines into synovial tissue and secrete a large number of pro-inflammatory cytokines to induce synovial inflammation. Moreover, RA-FLS can adhere to cartilage and release matrix metalloproteinase (MMPs) to degrade extracellular matrix and induce osteoclast activation and differentiation, which eventually results in bone erosion and joint destruction. Clinical studies showed that the levels of TNF-α and IL-1β in serum of RA patients were higher than those of normal people [21]. Normal SF cultured with synovial fluid of RA-FLS patients can secrete higher TNF-α and IL-1β, which indicates that activated RA-FLS can secrete high levels of inflammatory cytokines. Thus, IL-1β was added to SW982 to mimic the microenvironment of synovial fibroblasts in vitro, and osthole could inhibit IL-1β-induced proliferation and migration, and decrease the generation of MMP-1, MMP-3, MMP-13, IL-6 and TNF-α.

The NF-κB and MAPK signaling pathway is essential in regulating many cellular processes including inflammation, cell stress response, cell differentiation, cell division, cell proliferation, metabolism, motility and apoptosis. Moreover, synovial fibroblast targeting an NF-κB-blocking peptide improves synovial inflammation [22]. Andrographolide ameliorates TNFα-stimulated human RA synovial fibroblasts (RA-SFs) by inhabiting MAPK [23]. Using western blot, we found that osthole could suppress the NF-κB and MAPK signaling pathway in a dose-dependent manner. Oxidative stress is also involved in rheumatoid arthritis [24]. Although inhibition of oxidative stress was observed in the CIA model, whether osthole can ameliorate oxidative stress under IL-1β treatment and whether the antioxidative effect occurs earlier than anti-inflammation require further studies.

## Conclusion

In conclusion, it was found that osthole exerted a significant improvement effect on the CIA model. Osthole also inhibited IL-1β induced proliferation, migration, and the generation of MMPs and pro-inflammatory factors in vitro. The potential mechanism of osthole’s effect on synovial fibroblasts could be the modulation of the inflammation signaling pathway and anti-cellular stress. As a natural product without any significant side effects, osthole can be developed into a promising auxiliary drug for rheumatoid arthritis.

## Abbreviations

CIA: Collagen-induced arthritis
FBS: Fetal bovine serum
IL-1β: Interleukin-1β
IL-6: Interleukin-6
MMP: Matrix metalloproteinase
MTT: 3-(4,5-dimethyl-2-thiazolyl)-2,5-diphenyl-2-H-tetrazolium bromide
TNF-α: Tumor necrosis factor-α

## References

[1] Firestein GS (2003). Evolving concepts of rheumatoid arthritis. Nature.

[2] Terao C, Raychaudhuri S, Gregersen PK (2016). Recent advances in defining the genetic basis of rheumatoid arthritis. Annu Rev Genomics Hum Genet.

[3] Deane KD, Demoruelle MK, Kelmenson LB (2017). Genetic and environmental risk factors for rheumatoid arthritis. Best Pract Res Clin Rheumatol.

[4] Walsh MC, Takegahara N, Kim H (2018). Updating osteoimmunology: regulation of bone cells by innate and adaptive immunity. Nat Rev Rheumatol.

[5] Bartok B, Firestein GS (2010). Fibroblast-like synoviocytes: key effector cells in rheumatoid arthritis. Immunol Rev.

[6] Huber LC, Distler O, Tarner I (2006). Synovial fibroblasts: key players in rheumatoid arthritis. Rheumatology.

[7] Noss EH, Brenner MB (2008). The role and therapeutic implications of fibroblast-like synoviocytes in inflammation and cartilage erosion in rheumatoid arthritis. Immunol Rev.

[8] Jones DS, Jenney AP, Swantek JL (2017). Profiling drugs for rheumatoid arthritis that inhibit synovial fibroblast activation. Nat Chem Biol.

[9] Li Y, Li Y, Shi F (2018). Osthole attenuates right ventricular remodeling via decreased myocardial apoptosis and inflammation in monocrotaline-induced rats. Eur J Pharmacol.

[10] Bao Y, Meng X, Liu F (2018). Protective effects of osthole against inflammation induced by lipopolysaccharide in BV2 cells. Mol Med Rep.

[11] Liu PY, Chang DC, Lo YS (2018). Osthole induces human nasopharyngeal cancer cells apoptosis through Fas-Fas ligand and mitochondrial pathway. Environ Toxicol.

[12] Wang J, Fu Y, Wei Z (2017). Anti-asthmatic activity of osthole in an ovalbumin-induced asthma murine model. Resp Physiol Neuro.

[13] Duan J, Yang Y, Liu H (2016). Osthole ameliorates acute myocardial infarction in rats by decreasing the expression of inflammatory-related cytokines, diminishing MMP-2 expression and activating p-ERK. Int J Mol Med.

[14] Hua KF, Yang SM, Kao TY (2013). Osthole mitigates progressive IgA nephropathy by inhibiting reactive oxygen species generation and NF-κB/NLRP3 pathway. PLoS One.

[15] Kao SJ, Su JL, Chen CK (2012). Osthole inhibits the invasive ability of human lung adenocarcinoma cells via suppression of NF-κB-mediated matrix metalloproteinase-9 expression. Toxicol Appl Pharmacol.

[16] Alonzi T, Fattori E, Lazzaro D (1998). Interleukin 6 is required for the development of collagen-induced arthritis. J Exp Med.

[17] Walle LV, Van Opdenbosch N, Jacques P (2014). Negative regulation of the NLRP3 inflammasome by A20 protects against arthritis. Nature.

[18] Vuolteenaho K, Moilanen T, Hämäläinen M (2003). Regulation of nitric oxide production in osteoarthritic and rheumatoid cartilage. Role of endogenous IL-1 inhibitors. Scand J Rheumatol.

[19] Hu MM, Yang Q, Zhang J (2014). TRIM38 inhibits TNFα-and IL-1β-triggered NF-κB activation by mediating lysosome-dependent degradation of TAB2/3. P Natl Acad Sci USA.

[20] Yamanishi Y, Firestein GS (2001). Pathogenesis of rheumatoid arthritis: the role of synoviocytes. Rheum Dis Clin N Am.

[21] Casnici C, Lattuada D, Tonna N, et al. Optimized “in vitro” culture conditions for human rheumatoid arthritis synovial fibroblasts. Mediat Inflamm. 2014;2014:702057. 10.1155/2014/702057 PMC4235579 25548436

[22] You C, Zu J, Liu X (2018). Synovial fibroblast-targeting liposomes encapsulating an NF-κB-blocking peptide ameliorates zymosan-induced synovial inflammation. J Cell Mol Med.

[23] Li Z, Tan J, Wang L (2017). Andrographolide benefits rheumatoid arthritis via inhibiting MAPK pathways. Inflammation.

[24] Phull AR, Nasir B, ul Haq I (2017). Oxidative stress, consequences and ROS mediated cellular signaling in rheumatoid arthritis. Chem Biol Interact.

